# Purtscher-Like Retinopathy Associated With Acute Pancreatitis: A Case Report

**DOI:** 10.7759/cureus.73595

**Published:** 2024-11-13

**Authors:** Robert P VanHoy, Michael A Redmond

**Affiliations:** 1 Ophthalmology, Edward Via College of Osteopathic Medicine, Monroe, USA; 2 Ophthalmology, Louisiana Eye and Laser, Alexandria, USA

**Keywords:** acute pancreatitis, leukoembolization, purtscher flecken, purtscher-like retinopathy, purtscher retinopathy

## Abstract

Purtscher retinopathy is a type of ischemic retinopathy that can lead to a devastating visual prognosis. With no significantly proven treatment, preventing the condition by managing the causes and risk factors is the best way to preserve vision. This case report will focus on a patient with Purtscher-like retinopathy associated with acute pancreatitis, including exam findings, risk factors, and a discussion of treatment options.

## Introduction

Purtscher retinopathy (PR) can be caused by many things that subject the body to some type of stress. Recorded cases range from crush injury and preeclampsia to Valsalva maneuver and orbital area steroid injections [1]. This stress leads to increased levels of immune cells, which then embolize the small arteries in the retina, causing ischemia via a process called leukoembolization [1]. This blockage causes retinal ischemia and optic disk swelling, leading to a loss of vision [1]. Specifically, when acute pancreatitis (AP) is a cause, fats can embolize from pancreatic enzymes breaking down omental fats [1]. A study in the United Kingdom estimated the incidence of symptomatic PR to be 0.24 cases per million annually, with many cases thought to be asymptomatic [2]. Purtscher-like retinopathy (PLR) is clinically similar to PR but caused by non-traumatic stressors like AP [3,4]. The classical presentation of the PR fundus exam is Purtscher flecken (polygonal retinal whitening between the retinal arterioles and venules), retinal hemorrhages, and cotton-wool spots [5]. Diagnosing PR and PLR according to Miguel et al. requires the presence of three out of five diagnostic criteria: Purtscher flecken, retinal hemorrhages (in low to moderate number), cotton-wool spots (confined to the posterior pole), probable explanatory etiology (long bone fracture, AP, and renal failure), and complementary investigation compatible with diagnosis (elevated C5a level) [5]. AP causing PLR is especially concerning because it almost always presents bilaterally, whereas other causes present bilaterally only 60% of the time [2]. Treatment with high-dose corticosteroids over the first three days of symptom onset is thought to lead to a better visual outcome; however, this is not yet shown to be statistically significant [2]. Visual prognosis of PLR is usually poor with many of the changes being irreversible [3]. Because the prognosis of PLR is poor after the disease manifests, it is important to prevent triggering an enticing event. Specifically, many risk factors are associated with the development of AP, and many are modifiable [6]. Alcohol, smoking, and drugs like angiotensin-converting enzyme inhibitors, statins, and antidiabetic agents such as GLP-1 mimetics are well documented to be associated with the development of AP [6]. Food- or diet-induced AP is a less documented association but has been shown to possibly lower the threshold and work synergistically with other causes of AP [7]. A high-protein and -fat diet, together with alcohol use or gallstones, has been shown to lower the threshold for AP over time, increasing chances for AP over simply alcohol use or gallstones on their own [7]. Low body mass has also been associated with AP, especially in the setting of anorexia and bulimia nervosa, which can also cause chronic damage to the pancreas from oxidative stress [7]. Refeeding syndrome has been thought to be a cause of AP. Refeeding syndrome worsens gastric dilation and duodenal ileus caused by prolonged fasting, which causes retrograde pressure leading to reflux of duodenal contents into the pancreatic duct. This then leads to the activation of dormant pancreatic enzymes triggering AP [7]. The case below is of a young patient who develops PLR in the setting of AP. This case report highlights an example where the controversial treatment of PLR with high-dose intravenous steroids did not improve overall vision.

## Case presentation

**Figure 1:**
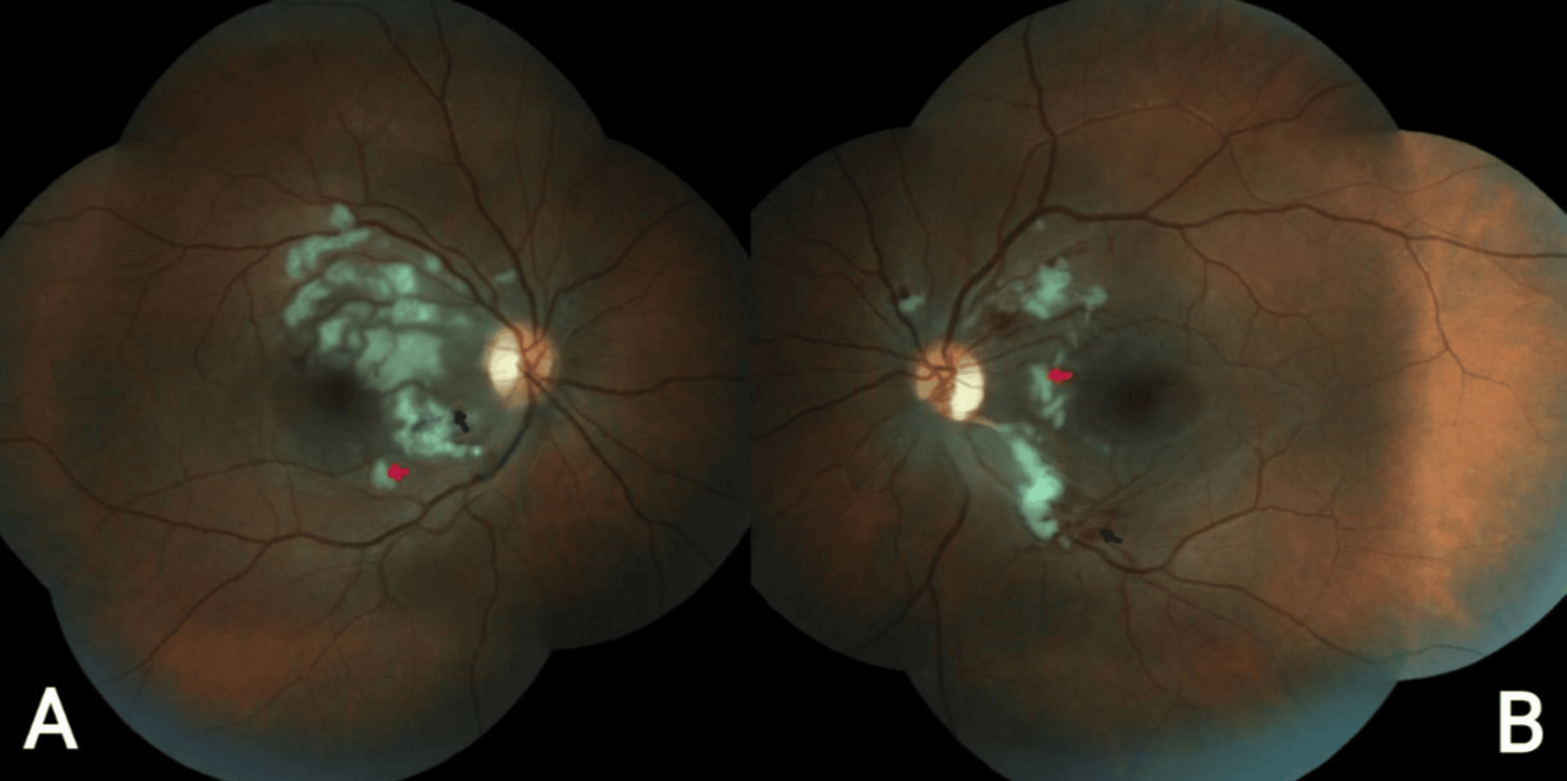
Fundus photo: (A) right eye (OD) fundus photo demonstrating intraretinal hemorrhages (black arrow) and cotton-wool spots (red arrow); (B) left eye (OS) fundus photo demonstrating intraretinal hemorrhages (black arrow) and cotton-wool spots (red arrow) OD: oculus dexter; OS: oculus sinister

**Figure 2:**
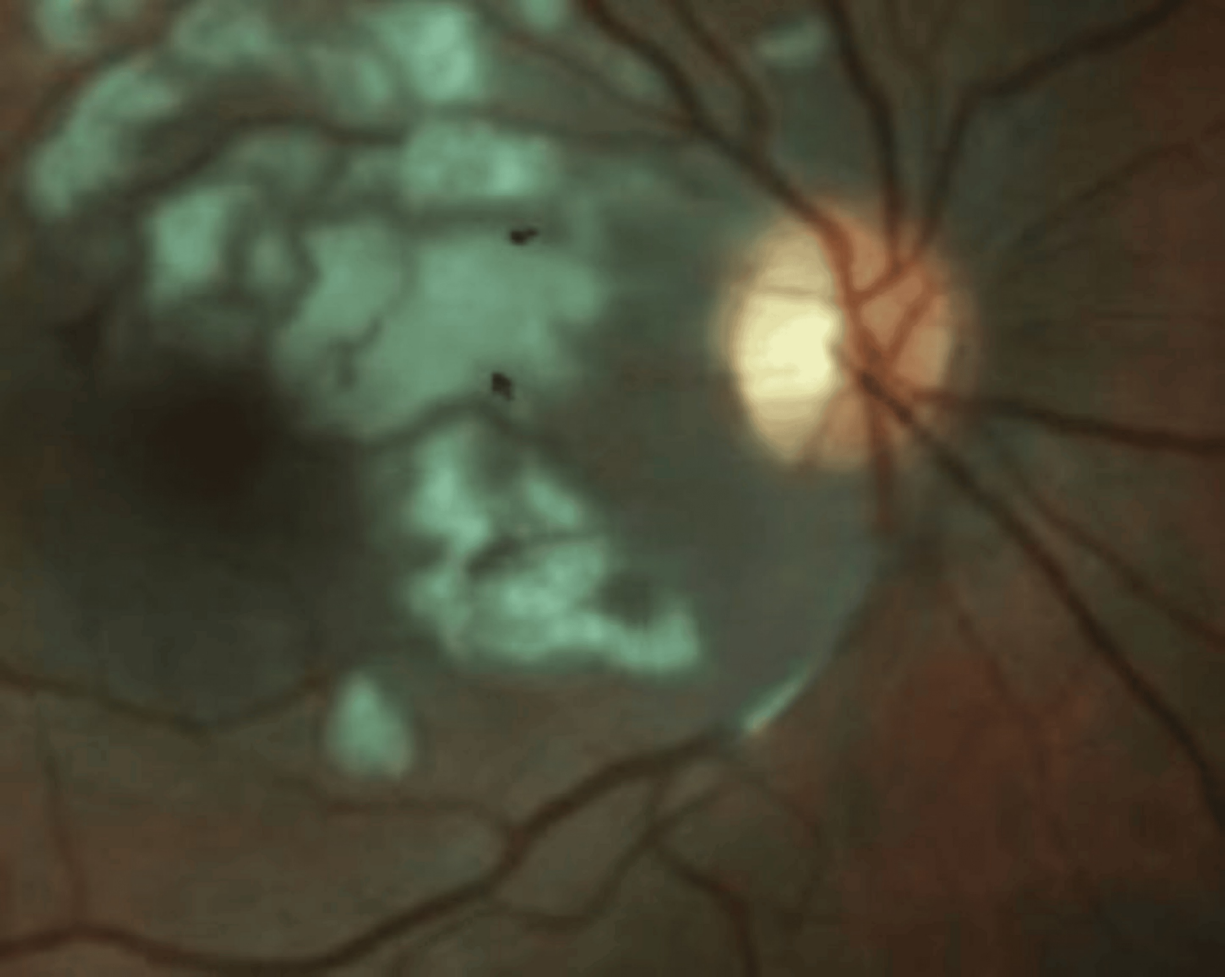
Purtscher flecken: fundus photo of the right eye showing polygonal retinal whitening between the retinal arterioles and venules demonstrating an example of Purtscher flecken (black arrows)

**Figure 3:**
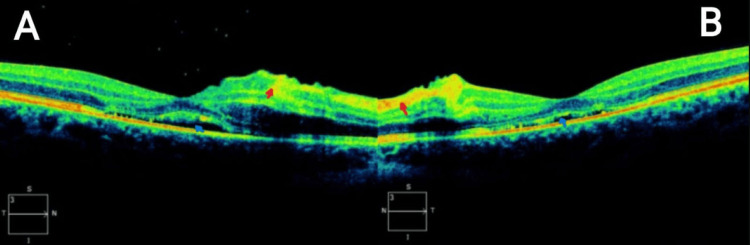
Optical coherence tomography (OCT) photo: (A) right eye (OD) OCT demonstrating subfoveal subretinal fluid (blue arrow) with hyperreflectivity in the inner retinal layers corresponding to cotton-wool spots (red arrow); (B) left eye (OS) OCT demonstrating a lesser degree of subfoveal subretinal fluid (blue arrow) with hyperreflectivity in the inner retinal layers corresponding to cotton-wool spots (red arrow) OD: oculus dexter; OS: oculus sinister

A 21-year-old woman was admitted to the hospital for AP and later discharged to outpatient ophthalmology for complaints of monocular blurry vision. When presenting to the ophthalmologist, along with blurry vision, she endorsed black dots, flashes of light, and double vision that had been affecting her since the day of her admission four days prior. The patient had a medical history of hypertension, anxiety, anorexia nervosa, and bulimia nervosa. She also stated she occasionally consumed liquor shots to help her sleep. The patient’s visual acuity was assessed as counting fingers at 2 m on the right and 20/70 on the left with best-corrected vision. Visual field by confrontation was diminished in the bilateral superotemporal and infratemporal quadrants. Intraocular pressures were within acceptable ranges, 15 on the right and 16 on the left. The slit lamp exam was normal in both eyes. Fundoscopic exam demonstrated bilateral peripapillary and posterior pole retinal whitening with intraretinal hemorrhages and cotton-wool spots. Fundus photos also demonstrated hallmarks of PLR with intraretinal hemorrhages, cotton-wool spots (Figure 1), and Purtscher flecken (Figure 2). Optical coherence tomography (OCT) photos demonstrated subfoveal subretinal fluid with hyperreflectivity in the inner retinal layers corresponding to cotton-wool spots (Figure 3). Based on the previous diagnosis of AP and the pathognomonic exam findings, the need for a fluorescein angiogram was deemed unnecessary, and the patient was diagnosed with PLR associated with AP. For treatment, the patient was admitted to the hospital to receive 1,000 mg IV methylprednisolone for three days. Her final visual acuity after treatment was counting fingers at 2 m in the right eye and 20/70 in the left. After being discharged, she was directed to see a retinal specialist for long-term follow-up to monitor progression and possible spontaneous visual recovery. The patient was followed by the specialist with little to no improvement attributed to retinal ischemia and tissue atrophy. The patient was then later lost to follow-up.

## Discussion

Currently, there is no standardized treatment of PLR. Several reports demonstrate the success of high-dose steroid treatment [8-10]. They cite the success due to steroid inhibition of the granulocyte adhesion and complement activation preventing leukoembolization. Additionally, steroids act to stabilize neural membranes and microvascular channels leading to the recovery of any neuronal fibers that have not been damaged beyond repair. However, prospective trials have not proven this to be true. A systematic review by Miguel et al. also demonstrated that there was no significant change in visual prognosis with IV high-dose steroids versus a patient that was simply observed. Topical non-steroidal anti-inflammatory drugs have been used for PLR and have been shown to help decrease retinal edema [11]. Papaverine hydrochloride has been used in a few cases to dilate arterioles to increase blood and oxygen flow in the retina, but the reproducibility and dosing of this treatment remain unclear [12].

Many treatments for PLR have been trialed with no preferred option. High-dose intravenous methylprednisolone has been shown to be the most reproducible treatment option, but like in our case, the outcomes vary. This may be due to the various inciting factors and presentations of the disease as well as the timing of the treatment. Further studies on treatments need to be performed to establish an optimal therapy for PLR.

## Conclusions

PLR associated with AP is a serious complication of AP and can cause long-lasting visual defects. To hasten the diagnosis, patients should be evaluated by an ophthalmologist when first presenting signs. Due to the lack of effective treatment, primary prevention by managing modifiable risk factors for AP remains the best way to preserve vision.
